# Association Between Risk of COVID-19 Infection in Nonimmune Individuals and COVID-19 Immunity in Their Family Members

**DOI:** 10.1001/jamainternmed.2021.5814

**Published:** 2021-10-11

**Authors:** Peter Nordström, Marcel Ballin, Anna Nordström

**Affiliations:** 1 Unit of Geriatric Medicine, Department of Community Medicine and Rehabilitation, Umeå University, Umeå, Sweden; 2 Section of Sustainable Health, Department of Public Health and Clinical Medicine, Umeå University, Umeå, Sweden; 3 School of Sport Sciences, UiT The Arctic University of Norway, Tromsø, Norway

## Abstract

**Importance:**

The association between COVID-19 immunity within families and the risk of infection in nonimmune family members is unknown.

**Objective:**

To investigate the association between risk of COVID-19 in nonimmune individuals and the number of their family members with known immunity acquired from a previous COVID-19 infection or full vaccination (2 vaccine doses).

**Design, Setting, and Participants:**

In this cohort study of data from nationwide registries in Sweden, all individuals who acquired immunity from either previous COVID-19 infection or full vaccination until May 26, 2021, were considered for inclusion. Each person with immunity was matched 1:1 to an individual without immunity from an identified cohort of individuals with families comprising 2 to 5 members.

**Exposures:**

Number of immune family members in each family on April 14, 2021 (index date), who acquired immunity from a previous COVID-19 infection or full vaccination (2 doses of the mRNA-1273, BNT162b2 mRNA, or ChAdOx1 nCoV-19 vaccine).

**Main Outcomes and Measures:**

Incident COVID-19 infection in nonimmune family members from April 15 to May 26, 2021.

**Results:**

A total of 1 789 728 individuals from 814 806 families were included in the analysis. Each family comprised 2 to 5 family members, with a mean (SD) age at baseline of 51.3 (19.5) years. During a mean (range) follow-up time of 26.3 (1-40) days, 88 797 of 1 549 989 (5.7%) nonimmune family members (mean [SD] age, 51.6 [17.7] years; 790 276 men [51.0%]) were diagnosed with COVID-19. There was an inverse dose-response association between the number of immune members in each family and the risk of incident COVID-19 infection in nonimmune family members. Nonimmune families with 1 immune family member had a 45% to 61% lower risk of contracting COVID-19 (hazard ratio [HR], 0.39-0.55; 95% CI, 0.37-0.61, *P* < .001). The risk reduction increased to 75% to 86% in families with 2 immune family members (HR, 0.14-0.25; 95% CI, 0.11-0.27; *P* < .001), 91% to 94% with 3 immune family members (HR, 0.06-0.09; 95% CI, 0.04-0.10; *P* < .001), and 97% with 4 immune family members (HR, 0.03; 95% CI, 0.02-0.05; *P* < .001). The results were similar for the outcome of COVID-19 infection that was severe enough to warrant a hospital stay.

**Conclusions and Relevance:**

In this cohort study, family members without immunity had a 45% to 97% lower risk of contracting COVID-19 as the number of immune family members increased. Vaccination is a key strategy for decreasing the transmission of the virus within families.

## Key Points

**Question:**

How is COVID-19 immunity within families associated with the risk for infection in family members without immunity?

**Findings:**

In this cohort study of 1 789 728 individuals from 814 806 families in Sweden, family members without immunity had a 45% to 97% lower risk of contracting COVID-19 as the number of immune family members increased.

**Meaning:**

These results suggest that COVID-19 vaccines play a key role in reducing the transmission of the virus within families, which likely has implications for herd immunity and pandemic control.

## Teaser

This cohort study examines the spread of COVID-19 infection within family members living in the same residence with different levels of immunity.

## Introduction

The COVID-19 pandemic has been a global health crisis of immense proportion,^1^ accounting for more than 175 million confirmed cases and 3.79 million deaths as of June 14, 2021.^2^ In response, vaccines against incident COVID-19 infection have been developed and evaluated at an unprecedented speed, showing an efficacy of about 70% to 95%.^3,4,5,6,7^ With mass vaccination campaigns occurring worldwide, the hope is that the resulting immunity in the population will not only prevent critical illness and death but also alleviate societal restrictions that are in place to reduce transmission, and thus aid in ending the pandemic. In Sweden, vaccinations were initially prioritized on the basis of identified risk factors for severe COVID-19 infection, including older age and certain medical conditions.^8^

Most of the global population has not yet been vaccinated and thus remains susceptible to infection.^2^ It is anticipated that most of the population in low-income countries will be unable to receive a vaccine in 2021, with current vaccination rates suggesting that completely inoculating 70% to 85% of the global population may take up to 5 years.^9^ Because SARS-CoV-2 is primarily spread through person-to-person contact,^10^ a family represents a high-risk setting of transmission.^11,12,13^ Studying the dynamics of family transmission can provide useful data on the extent to which acquired immunity within a family is associated with the risk of infection in nonimmune family members. This insight is important for making decisions about strategies for vaccination in low-income countries with limited vaccine supply.

In this nationwide cohort study, we used data from national registries in Sweden to investigate the association between risk of COVID-19 in nonimmune individuals and the number of their family members with known immunity from either a previous COVID-19 infection or full vaccination. We also investigated the difference in risks according to whether immunity was acquired from a previous infection, a single dose of vaccine, or full vaccination (2 vaccine doses).

## Methods

The present cohort study was approved by the Swedish Ethical Review Authority. Given the retrospective nature of the study, the requirement for obtaining participant informed consent was waived.

### Study Cohort

**Figure 1.**
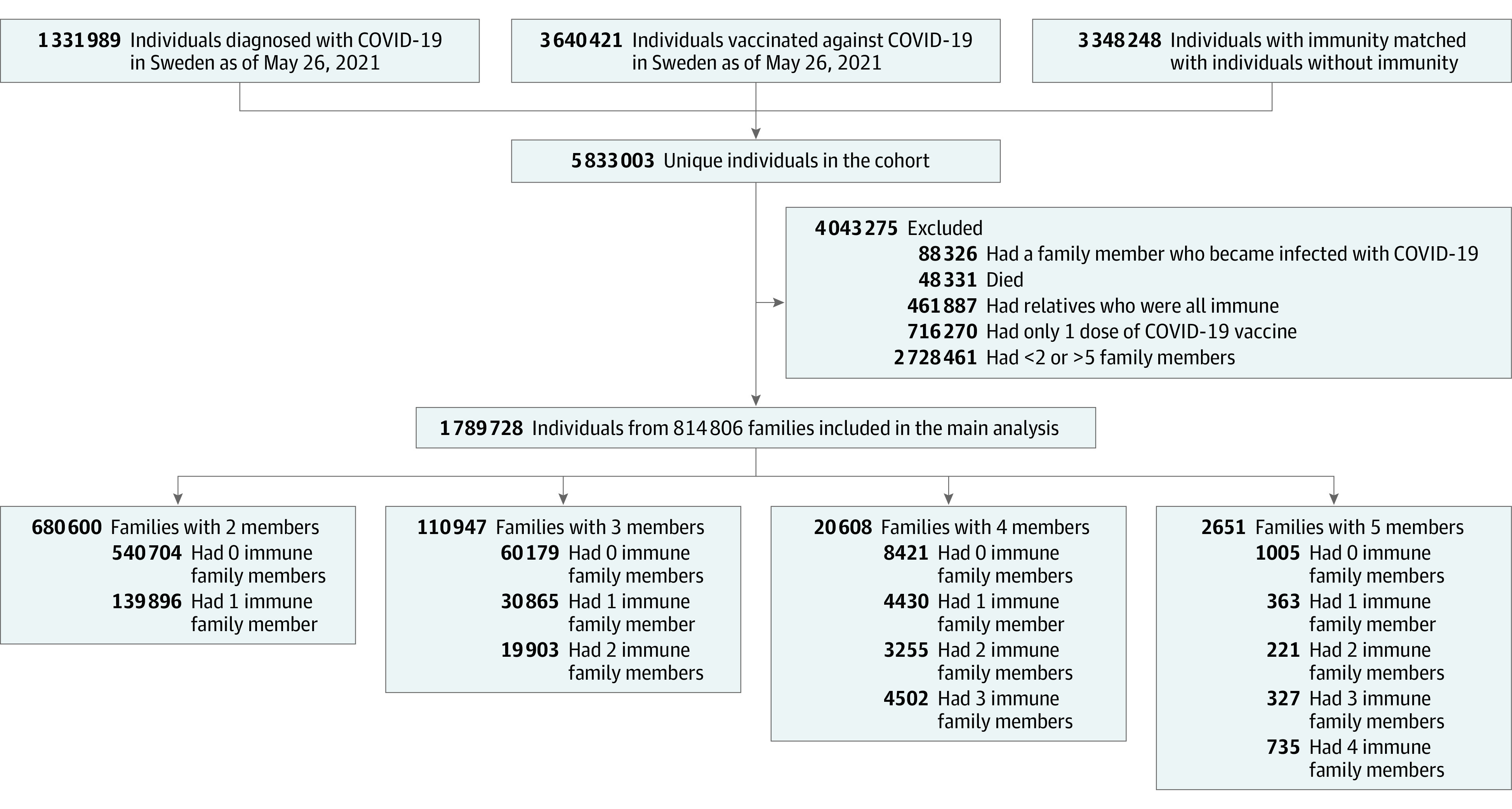
Description of the Selection of Families Included in the Main Analysis

The selection of the families included in this study is presented in Figure 1. We considered for inclusion all individuals who were diagnosed with COVID-19 (n = 1 331 989) and/or vaccinated against COVID-19 (n = 3 640 421) in Sweden until May 26, 2021. Each person with immunity was matched 1:1 to 3 348 248 individuals based on birth year, birth month, and municipality from the total population of Sweden using Statistics Sweden, the government agency that oversees statistical data.^14^ One nonimmune individual could then be matched to several individuals with a previous COVID-19 infection or vaccination. The matched individuals were not vaccinated against or diagnosed with COVID-19 at the date that the corresponding individuals were diagnosed with or vaccinated against COVID-19. Altogether, the total cohort consisted of 5 833 003 unique individuals. From this cohort, we identified 1 685 856 families, including those with 2 to 11 family members (n = 3 105 662). Families were defined as related individuals living at the same address and were identified using national registers that are managed by Statistics Sweden.^14^ Data on individuals who were vaccinated against or infected with SARS-CoV-2 were collected from the Swedish National Vaccination Register and SmiNet database, respectively, both of which are managed by the Public Health Agency of Sweden.^15,16^ All health care practitioners in Sweden are obliged by Swedish law to report to these registers, ensuring a 100% coverage of the Swedish population. The main outcome of incident COVID-19 infection during follow-up was collected in nonimmune family members.

In all analyses, we set April 1, 2021, as the baseline date and April 14, 2021, as the index date. Families were excluded if any family members became infected with COVID-19 between the baseline and index dates (n = 88 326); in this way, infections that were contracted close to the index date would not alter the risk of infections after the index date. Individuals who died before the index date (n = 48 331) were also excluded. After excluding families with more than 5 members (n = 1120) because of small sample sizes, we were able to retain 2 957 891 individuals from 1 599 464 families.

For the main analysis, we further excluded individuals with only a single dose of vaccine before the index date (n = 716 270). Only nonimmune members of each family were included in the statistical models with the outcome of incident COVID-19 infection (the distribution is presented in Figure 1).

### Immunity, Exposure, and Outcome

For the main analysis, we specified that immunity was acquired from either a COVID-19 infection before the baseline date or full vaccination, the receipt of 2 doses of the mRNA-1273 (Moderna), BNT162b2 mRNA (Pfizer-BioNTech), or ChAdOx1 nCoV-19 (Oxford/AstraZeneca) vaccine before the index date. A COVID-19 infection was confirmed by polymerase chain reaction in 94.4% of the cases and by sequencing in 4.8% of the cases, according to SmiNet.^16^

The main exposure was the number of individuals with immunity within each family. The outcome was incident COVID-19 infection in nonimmune family members from April 15 to May 26, 2021. For the main exposure of immunity from a previous infection or full vaccination, we also evaluated incident COVID-19 infections that were severe enough to result in a hospital stay. These hospitalization data were traced in the National Inpatient Register and the National Outpatient Register, which are overseen by the Swedish National Board of Health and Welfare, using the *International Statistical Classification of Diseases and Related Health Problems, Tenth Revision* code U071. All analyses were stratified by the total number of family members in each family (2 to 5).

### Covariates

Covariates were selected based on those in a previous study of the risk factors for COVID-19 infection in a similar cohort.^8^ Data on selected diagnoses were obtained from the National Inpatient Register since 1998 and from the National Outpatient Register since 2001 using *International Statistical Classification of Diseases and Related Health Problems, Tenth Revision* codes. Both of these registers contain complete data for specialty health care services provided in Sweden for the years selected. From Statistics Sweden, we obtained information on whether the individuals were born in Sweden as well as their birth year, birth month, and sex.^14^ Statistics Sweden also provided information on highest educational level, total income, and early retirement pension for 2019. Data on educational level were missing for 3.3% of the individuals, and early retirement pension information was missing for 6.2% of the individuals.

### Statistical Analysis

Time to event for incident COVID-19 infection by the number of immune family members was illustrated with cumulative incidence curves and corresponding 95% CIs and was estimated with the Kaplan-Meier method. To compare the risk of COVID-19 infection across the number of immune family members in each family, we used Cox proportional hazards regression models for unconditional samples. To adjust for clustering within families, we used the VCE command and cluster option in the Stata software (StataCorp LLC) to calculate robust SE. For nonimmune family members, follow-up time was counted from April 15, 2021, until the date of confirmed COVID-19 infection, a first dose of vaccine, death, or May 26, 2021, whichever occurred first. Thus, we collected outcomes in all nonimmune family members, and follow-up time was not terminated in nonimmune family members because of a COVID-19 infection in another member of the same family.

Trends were investigated by including categorical variables as numbers in the Cox proportional hazards regression models. The proportional hazards assumption was checked using log-minus-log plots and was not violated. Families with no immunity at index date were the reference group in all models, and regression analyses were performed separately for families with 2, 3, 4, or 5 family members. The first model was adjusted for age. The second model included sex, educational level (6 categories), early retirement pension, total income (in euros), whether individuals were born in Sweden, and baseline diagnosis.

In the first sensitivity analysis, immunity was acquired from a previous infection, and individuals with a first dose of vaccine before the index date were excluded. In the second sensitivity analysis, immunity was acquired from a single dose of vaccine, and individuals with 2 doses of vaccine or an infection before the index date were excluded. Because of the fewer number of immune family members in the second sensitivity analysis, families with 5 members were excluded. It was not possible to do a sensitivity analysis that was limited to people who had received 2 doses of vaccine because too few persons had received both vaccines by the April 1, 2021, baseline date.

All analyses were performed in Stata, version 16.1 for Mac. A 2-sided *P* < .05 or a hazard ratio (HR) with 95% CI less than 1 was considered significant.

## Results

A total of 1 789 728 individuals from 814 806 families were included in the main analysis. Each family comprised 2 to 5 family members, with a mean (SD) age at baseline of 51.3 (19.5) years. The most common family constellation was a 2-member family with no immunity (n = 1 081 408). The most uncommon family constellation was a 5-member family whose immunity (from a previous infection or vaccination) was present in 4 of 5 family members (n = 735). Baseline characteristics for nonimmune individuals (n = 1 549 989; 790 276 men [51.0%] and 759 713 women [49.0%]) are presented in Table 1, for whom a clear trend can be seen toward lower mean (SD) age (51.6 [17.7] years vs 27.3 [15.5] years for 4 immune family members; *P* < .001), fewer medical diagnoses (myocardial infarction: 30 554 [2.0%] vs 3 [0.4%]; *P* < .001), lower mean (SD) income (€29 247/US $34 671 [€80 770/US $95 750] vs €9205/US $10 912 [€14 242/US $16 883]; *P* < .001), and a lower proportion of individuals who were born in Sweden (1 267 527 [81.8%] vs 534 [72.7%]; *P* < .001) as the number of immune family members increased.

**Table 1.** Baseline Characteristics of Nonimmune Individuals With Immunity Acquired From a Previous COVID-19 Infection or Full Vaccination

Characteristic	No. (%)					
	All nonimmune individuals (n = 1 549 989)	No. of individuals with immunity in each family at baseline^a^				
		0 Immune family members (n = 1 300 654)	1 Immune family member (n = 216 368)	2 Immune family members (n = 27 076)	3 Immune family members (n = 5156)	4 Immune family members (n = 735)
Age, mean (SD), y	51.6 (17.7)	52.9 (17.3)	46.8 (18.0)	36.1 (18.5)	31.2 (17.0)	27.3 (15.5)
Female sex	759 713 (49.0)	654 930 (50.4)	90 339 (41.8)	11 787 (43.5)	2305 (44.7)	352 (47.9)
Male sex	790 276 (51.0)	645 724 (49.6)	126 029 (58.2)	15 239 (56.5)	2851 (55.3)	383 (52.1)
Highest educational level^b^						
Elementary school						
<9 y	63 702 (4.1)	55 683 (4.3)	7236 (3.3)	642 (2.4)	123 (2.4)	18 (2.4)
9 y	190 517 (12.3)	156 195 (12.0)	28 870 (13.3)	4332 (16.0)	969 (18.8)	151 (20.5)
Secondary school						
2 y	365 575 (23.6)	314 012 (24.1)	47 238 (21.8)	3794 (14.0)	487 (9.4)	44 (6.0)
>2 y	273 820 (17.7)	226 490 (17.4)	40 760 (18.8)	5332 (19.7)	1074 (20.8)	164 (22.3)
University education	542 257 (35.0)	462 885 (35.6)	71 105 (32.9)	7025 (25.9)	1115 (21.6)	127 (17.3)
Unknown^b^	114 118 (7.4)	85 389 (6.6)	21 159 (9.8)	5951 (22.0)	1388 (26.9)	231 (31.4)
Total income, mean (SD), €^b^^,^^c^	29 247 (80 770)	29 442 (70 542)	29 219 (123 477)	22 787 (111 364)	17 482 (22 741)	9205 (14 242)
Early retirement pension	61 589 (4.0)	53 754 (4.1)	7016 (3.2)	707 (2.6)	95 (1.8)	17 (2.3)
Born in Sweden	1 267 527 (81.8)	1 067 805 (82.1)	173 731 (80.3)	21 451 (79.2)	4006 (77.7)	534 (72.7)
Diagnosis						
Myocardial infarction	30 554 (2.0)	26 947 (2.1)	3381 (1.6)	191 (0.7)	32 (0.6)	3 (0.4)
Stroke	20 225 (1.3)	17 877 (1.4)	2207 (1.0)	117 (0.4)	21 (0.4)	3 (0.4)
Diabetes	119 895 (7.7)	103 660 (8.0)	14 757 (6.8)	1244 (4.6)	205 (4.0)	29 (3.9)
Hypertension	416 070 (26.8)	365 978 (28.1)	46 501 (21.5)	3152 (11.6)	395 (7.7)	44 (6.0)
Kidney failure	12 040 (0.8)	10 443 (0.8)	1458 (0.7)	116 (0.4)	22 (0.4)	1 (0.1)
COPD	13 597 (0.9)	12 179 (0.9)	1313 (0.6)	91 (0.3)	12 (0.2)	2 (0.3)
Cancer	63 272 (4.1)	56 467 (4.3)	6323 (2.9)	425 (1.6)	51 (1.0)	6 (0.8)

Abbreviation: COPD, chronic obstructive pulmonary disease.

^a^ Immunity was acquired from either a previous COVID-19 infection or full vaccination.

^b^ Education and income data were registered for individuals who were born earlier than October 2005. Unknown indicates that no information was found in the registers.

^c^ As of September 9, 2021, 1 Euro is equal to 1.18 US dollars.

### Immune Family Members and the Risk of COVID-19 in Nonimmune Family Members

**Figure 2.**
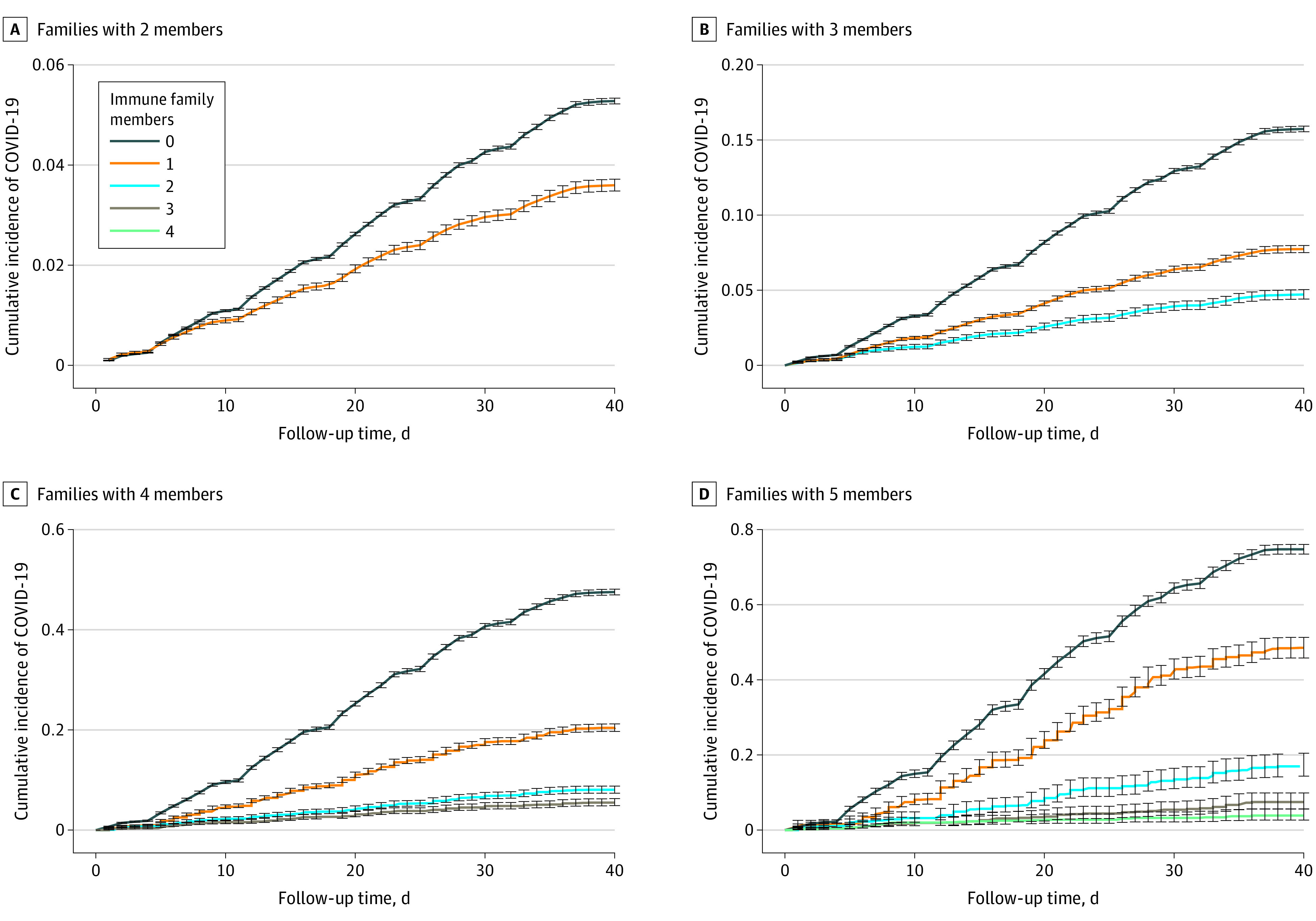
Risk of COVID-19 Infection in Families With 2 to 5 Members Time to event for the outcome of incident COVID-19 infection was illustrated based on the number of immune family members, using cumulative incidence curves with 95% CIs (error bars) and estimated using the Kaplan-Meier method. Immunity was acquired from either a previous COVID-19 infection or full vaccination.

During a total follow-up time of 111 454 years, we found that 88 797 of 1 549 989 nonimmune individuals (5.7%) were diagnosed with COVID-19 during a mean (range) follow-up time of 26.3 (1-40) days. There was a significant inverse dose-response association between the number of immune family members and the risk of incident COVID-19 infection in nonimmune family members (Figure 2) regardless of family size. Thus, in families with 1 immune family member, nonimmune family members had a 45% to 61% lower risk of contracting COVID-19 regardless of family size (HR, 0.39-0.55; 95% CI, 0.37-0.61; *P* < .001 for all). In families with 2 immune family members, nonimmune family members had a 75% to 86% lower risk (HR, 0.14-0.25; 95% CI, 0.11-0.27; *P* < .001 for all), which was further reduced to 91% to 94% in families with 3 immune family members (HR, 0.06-0.09; 95% CI, 0.04-0.10; *P* < .001 for all). In families with 5 members, among whom 4 were immune, the remaining nonimmune family member had a 97% lower risk (HR, 0.03; 95% CI, 0.02-0.05; *P* < .001). Adjustment for covariates did not change the associations (Table 2).

**Table 2.** Risk of COVID-19 Infection in Nonimmune Family Members

No. of individuals with immunity in each family	No. of nonimmune individuals at risk	Incident COVID-19 infection, No. (%)	HR (95% CI)^a^	
			Model 1^b^	Model 2^c^
**Families with 2 members**				
0	1 081 408	35 165 (3.3)	1 [Reference]	1 [Reference]
1	139 896	3714 (2.7)	0.56 (0.54-0.58)	0.55 (0.53-0.57)
**Families with 3 members**				
0	180 537	23 516 (13.0)	1 [Reference]	1 [Reference]
1	61 730	3914 (6.3)	0.56 (0.54-0.58)	0.55 (0.54-0.57)
2	19 903	834 (4.2)	0.26 (0.24-0.28)	0.25 (0.23-0.27)
**Families with 4 members**				
0	33 684	14 268 (42.4)	1 [Reference]	1 [Reference]
1	13 290	2369 (17.8)	0.38 (0.36-0.41)	0.39 (0.37-0.42)
2	6510	468 (7.2)	0.14 (0.12-0.16)	0.14 (0.13-0.16)
3	4502	231 (5.1)	0.09 (0.08-0.10)	0.09 (0.08-0.10)
**Families with 5 members**				
0	5025	3505 (69.8)	1 [Reference]	1 [Reference]
1	1452	638 (43.9)	0.52 (0.44-0.60)	0.52 (0.45-0.61)
2	663	103 (15.5)	0.15 (0.11-0.20)	0.15 (0.11-0.20)
3	654	45 (6.9)	0.06 (0.04-0.08)	0.06 (0.04-0.09)
4	735	27 (3.7)	0.03 (0.02-0.05)	0.03 (0.02-0.05)

Abbreviation: HR, hazard ratio.

^a^ Cox proportional hazards regression models were used to calculate HRs, with the number of immune members in each family as the exposure and COVID-19 infection in nonimmune family members as the outcome. Analyses were performed separately for families with 2 to 5 members, using families with no immunity as the reference. Immunity was acquired from either a previous COVID-19 infection or full vaccination.

^b^ Adjusted for age.

^c^ Adjusted for age, sex, educational level, total income, early retirement pension, whether born in Sweden, and baseline diagnosis.

The results were similar for the outcome of COVID-19 infection that was severe enough to result in a hospital stay (Table 3). For example, in families with 3 members, among whom 2 were immune, the remaining nonimmune family member had an 80% lower risk (HR, 0.20; 95% CI, 0.10-0.43; *P* < .001).

**Table 3.** Risk of Severe COVID-19 Infection in Nonimmune Family Members

No. of individuals with immunity in each family	No. of nonimmune individuals at risk	Incident COVID-19 infection, No. (%)	HR (95% CI)^a^
**Families with 2 members**			
0	1 081 408	803 (0.7)	1 [Reference]
1	139 896	101 (0.7)	0.88 (0.71-1.08)
**Families with 3 members**			
0	180 537	340 (0.19)	1 [Reference]
1	61 730	61 (0.10)	0.50 (0.38-0.66)
2	19 903	7 (0.04)	0.20 (0.10-0.43)
**Families with 4 members**			
0	33 684	164 (0.49)	1 [Reference]
1	13 290	32 (0.24)	0.45 (0.31-0.67)
2	6510	4 (0.06)	0.11 (0.04-0.30)
3	4502	1 (0.02)	0.05 (0.01-0.36)

Abbreviation: HR, hazard ratio.

^a^ Cox proportional hazards regression models were used to calculate HR, with the number of immune family members as exposure and COVID-19 infection that was severe enough to result in a hospital stay in nonimmune family members as the outcome. Analyses were performed separately for families with 2 to 4 members, using families with no immunity as the reference. Immunity was acquired from either a previous COVID-19 infection or full vaccination. The regression models were adjusted for age.

### Sensitivity Analyses

The cohort in the first sensitivity analysis had similar characteristics at baseline as the cohort in the main analysis (eTable 1 in the Supplement). The results of the first sensitivity analysis showed a similar pattern and risk reductions as in the main analysis. For example, in families with 3 members, among whom 2 were immune, the remaining nonimmune family member had a 78% lower risk (HR, 0.22; 95% CI, 0.21-0.24; *P* < .001) (eTable 2 in the Supplement). The characteristics of the second sensitivity analysis cohort are presented in eTable 3 in the Supplement. As in the first sensitivity analysis, the results of the second sensitivity analysis are similar to those of the main analysis (eTable 4 in the Supplement). For example, in families with 3 members, among whom 2 were immune, the remaining nonimmune family member had a 66% lower risk (HR, 0.34; 95% CI, 0.29-0.39; *P* < .001).

## Discussion

In this nationwide cohort study of more than 0.8 million Swedish families, nonimmune family members had a 45% to 97% lower risk of COVID-19 that was in line with the increasing number of immune family members. The benefits were similar regardless of whether immunity was acquired from a previous infection, a single dose of vaccine, or full vaccination. These findings suggest that vaccines play a key role in reducing the transmission of the virus within families, which likely has implications for herd immunity and pandemic control.

This study found a dose-response association between the number of immune family members and the risk of COVID-19 in nonimmune family members. In families with only 1 immune family member, the remaining nonimmune family members were at a considerably lower risk of contracting COVID-19 (in the range of 45% to 61%) regardless of family size. Protection became more pronounced as the number of immune family members increased. In families with 2 immune family members, nonimmune family members had up to 86% lower risk, and in families with 3 or 4 immune family members, the nonimmune family members had 91% to 97% lower risk of contracting COVID-19. The likely reason for the higher relative protection of the last nonimmune family member in larger vs smaller families is that the analyses were stratified by family size. Thus, although the absolute risk of infection was associated with the number of nonimmune relatives in each family, the relative risk reduction was much higher in larger families. For example, the absolute risk of infection in the last immune family member was 3% to 5% in families with 4 or more members. These findings also suggest that the absolute risk of infection is dependent on the number of nonimmune members in each family.

Previous studies have reported that a single dose of vaccine is highly effective protection against infection, critical illness, and death,^17,18,19,20^ but the extent to which a single dose of vaccine can control transmission of the virus within families has been unknown thus far. In this study, we found that the benefit of immunity (ie, lowered risk of transmission within families) acquired from a single dose was similar to the benefit of immunity from full vaccination or a previous infection. This knowledge may be particularly valuable for low-income countries in which most people are unlikely to receive the vaccines in the near future.^9^ However, the results and conclusions of the present study, including these single-dose findings, apply only to the Alpha variant of SARS-CoV-2, which caused more than 95% of all COVID-19 cases at the time of this study’s follow-up.^21^ Reports have suggested that recipients of a single dose may have less protection against the Beta and Delta variants, and these variants appear to be more transmissible.^22,23^ For instance, a single dose of the BNT162b2 and ChAdOx1 nCoV-19 vaccines has been associated with a modest protection (approximately 30%) against the Delta variant,^24,25^ which seems to be the dominating variant in the most recent wave of the pandemic. Although the results of this study indicate similar benefits of immunity from a single vaccine dose and full vaccination, the evidence on emerging variants may promote full vaccination.

### Strengths and Limitations

This study has several strengths. First, it had a unique design that enabled the examination of families with different proportions of immunity. We also performed a comprehensive set of analyses to test whether the associations found differed according to whether immunity was acquired from contracting COVID-19 infection, from a single dose of vaccine, or from full vaccination. Second, the study included a large nationwide population of more than 0.8 million families, including approximately 1.8 million individuals. This size increases the external validity and generalizability of the findings to other nations.

The study also has some limitations. Although we adjusted the analyses for a set of covariates, based on the results of a previous study^8^ in a similar population with no changes in the regression estimates, other covariates could be factors in the associations found. In addition, the number of incident cases was rather low in the sensitivity analyses of the largest families. We also did not have enough participants who had received full (2 doses) vaccinations to test the impact of this subgroup alone.

## Conclusions

This nationwide cohort study showed that individuals without COVID-19 immunity had a 45% to 97% lower risk of infection that was in line with the increase in the number of immune family members. Similar results were found regardless of whether immunity was acquired from a previous infection, a single dose of vaccine, or full vaccination. These findings suggest that vaccines are associated with a reduction in the transmission of the SARS-CoV-2 virus within families, which likely has implications for herd immunity and pandemic control. However, caution is warranted given the emerging variants of concern, which appear more transmissible and may be less sensitive to a single dose of vaccine.

## Notes

Supplement. **eTable 1.** Baseline Characteristics When Immunity Was Defined as Previous Infection **eTable 2.** Risk of Covid-19 Infection in Families with Two-Five Members **eTable 3.** Baseline Characteristics When Immunity Was Defined as 1 Dose of Vaccine **eTable 4.** Risk of Covid-19 Infection in Families with Two-Four Members
