# Supplementary material for: Association Between Risk of COVID-19 Infection in Nonimmune Individuals and COVID-19 Immunity in Their Family Members
Source: JAMA Intern Med. 2021 Oct 11;181(12):1–8. doi: 10.1001/jamainternmed.2021.5814 (PMC8506298; doi:10.1001/jamainternmed.2021.5814)
Supplement: Supplement. — eTable 1. Baseline Characteristics When Immunity Was Defined as Previous Infection eTable 2. Risk of Covid-19 Infection in Families with Two-Five Members eTable 3. Baseline Characteristics When Immunity Was Defined as 1 Dose of Vaccine eTable 4. Risk of Covid-19 Infection in Families with Two-Four Members [file jamainternmed-e215814-s001.pdf]

Supplemental Online Content

Nordström P, Ballin M, Nordström A. Association Between risk of COVID-19 infection in nonimmune individuals and COVID-19 immunity in their family members. *JAMA Intern Med*. Published online October 11, 2021. doi:10.1001/ jamainternmed.2021.5814

eTable 1. Baseline Characteristics When Immunity Was Defined as Previous Infection
eTable 2. Risk of Covid-19 Infection in Families with Two-Five Members.
eTable 3. Baseline Characteristics When Immunity Was Defined as 1 Dose of Vaccine
eTable 4. Risk of Covid-19 Infection in Families with Two-Four Members.

This supplemental material has been provided by the authors to give readers additional information about their work.

© 2021 Nordström P, et al. *JAMA Internal Medicine.*

eTable 1. Baseline Characteristics When Immunity Was Defined as Previous Infection. Baseline characteristics for individuals with no immunity (N=1,430,339) in families with 2-5 family members. Data is presented based on number of immune individuals in each family at baseline. Immunity was defined as previous Covid-19 infection.

	**All families**	**Characteristics based on number of individuals with immunity in each family at baseline**				
	1,430,339	0 immune family members (N=1,262,464)	1 immune family member (N=140,707)	2 immune family members (N=21,747)	3 immune family members (N=4,721)	4 immune family members (N=700)
**Age, mean, SD**	50.8±17.6	51.9±17.3	44.8±17.1	35.1±18.3	31.0±17.0	27.4±15.6
Range	1.4-104.2	1.4-104.2	1.3-100.8	1.4-85.6	2.6-77.0	2.2-74.0
**Female sex , N (%)**	713,037 (49.9%)	633,630 (50.2%)	66,827 (47.5%)	10,098 (46.4%)	2,149 (45.5%)	333 (47.6%)
**Highest education, N (%)**						
Elementary school < 9yrs	55,704 (3.9%)	50,692 (4.0%)	4,338 (3.1%)	543 (2.5%)	113 (2.4%)	18 (2.6%)
Elementary school 9yrs	175,403 (12.3%)	153,390 (12.2%)	17,578 (12.5%)	3,406 (15.7%)	887 (18.8%)	142 (20.3%)
Secondary school, 2 yrs	329,821 (23.1%)	299,897 (23.8%)	26,758 (19.0%)	2,698 (12.4%)	427 (9.0%)	41 (5.9%)
Secondary school, >2 yrs	255,095 (17.8%)	222,313 (17.6%)	27,357 (19.4%)	4,280 (19.7%)	989 (20.9%)	156 (22.3%)
University education	504,084 (35.2%)	447,584 (35.5%)	49,674 (35.3%)	5,677 (26.1%)	1,026 (21.7%)	123 (17.6%)
Unknown[a]	110,232 (7.7%)	88,588 (7.0%)	15,002 (10.7%)	5,143 (23.6%)	1,279 (27.1%)	220 (31.4%)
**Income (Euro), mean, SD**	23,607±85,786	29,365±70,985	30,313±148,764	22,849±123,736	17,340±22,807	13,283±18,575
**Early retirement pension, N (%)**	57,505 (4.0%)	52,720 (4.2%)	4,213 (3.0%)	474 (2.2%)	83 (1.8%)	15 (2.1%)
**Born in Sweden, N (%)**	1,163,140 (81.3%)	1,032,526 (81.8%)	109,516 (77.8%)	16,944 (77,9%)	3,648 (77.3%)	506 (72.3%)
**Diagnoses, N (%)**						
Myocardial infarction	26,236 (1.8%)	24,483 (1.9%)	1,584 (1.1%)	139 (0.6%)	26 (0.6%)	4 (0.6%)
Stroke	17,352 (1.2%)	16,252 (1.3%)	996 (0.7%)	81 (0.4%)	20 (0.4%)	3 (0.4%)
Diabetes	106,335 (7.4%)	97,132 (7.7%)	8,093 (5.8%)	904 (4.2%)	179 (3.8%)	27 (3.9%)
Hypertension	365,753 (25.6%)	338,741 (26.8%)	24,383 (17.3%)	2,238 (10.3%)	349 (7.4%)	42 (6.0%)
Kidney failure	10,468 (0.7%)	9,623 (0.8%)	738 (0.5%)	84 (0.4%)	22 (0.5%)	1 (0.1%)
COPD[#]	11,662 (0.8%)	10,990 (0.9%)	591 (0.4%)	67 (0.3%)	12 (0.3%)	2 (0.3%)
Cancer	54,834 (3.8%)	51,186 (4.1%)	3,301 (2.3%)	299 (1.4%)	42 (0.9%)	6 (0.9%)

[a]Education and income were registered for individuals born earlier than October 2005. SD = standard deviation.

[#] Chronic obstructive pulmonary disease

© 2021 Nordström P, et al. *JAMA Internal Medicine.*

eTable 2. Risk of Covid-19 Infection in Families with Two-Five Members. Cox regression was used to calculate hazard ratios (HR) with the number of immune members in each family as exposure, and the outcome of Covid-19 infection in non-immune family members. Analyses were performed separately for families with 2-5 members, using families with no immunity as reference. Immunity was defined as a previous infection.

**Families with two relatives**		Model 1#		Model 2*	
Number of individuals with immunity in each family	Incident Covid-19 cases, (%)	HR	95% CI	HR	95% CI
None (N=1,004,018)	36,450 (3.5%)	1	(ref)	1	(ref)
One (N=83,530)	2,258 (2.7%)	0.48	0.46-0.50	0.47	0.45-0.49

**Families with three relatives**		Model 1#		Model 2*	
Number of individuals with immunity in each family	Incident Covid-19 cases, (%)	HR	95% CI	HR	95% CI
None (N=181,437)	24,258 (13.4%)	1	(ref)	1	(ref)
One (N=45,968)	2,558 (5.6%)	0.44	0.42-0.46	0.43	0.41-0.45
Two (N=15,962)	643 (4.0%)	0.23	0.21-0.25	0.22	0.21-0.24

**Families with four relatives**		Model 1#		Model 2*	
Number of individuals with immunity in each family	Incident Covid-19 cases, (%)	HR	95% CI	HR	95% CI
None (N=33,944)	14,490 (42.7%)	1	(ref)	1	(ref)
One (N=10,161)	1,580 (15.5%)	0.33	0.31-0.35	0.33	0.32-0.35
Two (N=5,266)	365 (6.9%)	0.13	0.12-0.15	0.13	0.12-0.15
Three (N=4,131)	220 (5.3%)	0.09	0.08-0.10	0.09	0.08-0.10

**Families with five relatives**		Model 1#		Model 2*	
Number of individuals with immunity in each family	Incident Covid-19 cases, (%)	HR	95% CI	HR	95% CI
None (N=5,065)	3,529 (69.7%)	1	(ref)	1	(ref)
One (N=1,048)	416 (39.7%)	0.45	0.41-0.50	0.45	0.41-0.50
Two (N=519)	73 (14.1%)	0.14	0.11-0.17	0.14	0.11-0.17
Three (N=590)	39 (6.6%)	0.06	0.04-0.08	0.06	0.04-0.08
Four (N=700)	22 (3.1%)	0.03	0.02-0.04	0.03	0.02-0.04

© 2021 Nordström P, et al. *JAMA Internal Medicine.*

eTable 3. Baseline Characteristics When Immunity Was Defined as 1 Dose of Vaccine. Baseline characteristics for individuals with no immunity (N=1,453,545) in families with 2-4 family members. Data is presented based on number of immune individuals in each family at baseline. Immunity was defined as a single dose of any vaccine.

	**All families**	**Characteristics based on number of individuals with immunity in each family at baseline**			
		0 immune family members	1 immune family member	2 immune family members	3 immune family members
	(N=1,453,545)	(N=1,291,760)	(N=157,148)	(N=4,493)	(N=144)
**Age, mean, SD**	52.2±17.4	51.7±17.3	56.0±17.9	39.4±16.3	23.4±12.8
Range	1.4-104.2	1.4-104.2	1.3-99.0	3.0-86.7	9.9-68.3
**Female sex , N (%)**	732,782 (50.4%)	649,534 (50.3%)	81,585 (51.9%)	1,602 (35.7%)	61 (42.4%)
**Highest education, N (%)**					
Elementary school < 9yrs	59,340 (4.1%)	51,667 (4.0%)	7597 (4.8%)	72 (1.6%)	4 (2.8%)
Elementary school 9yrs	177,413 (12.2%)	156,534 (12.1%)	20171 (12.8%)	682 (15.2%)	26 (18.1%)
Secondary school, 2 yrs	348,428 (24.0%)	304,717 (23.6%)	42862 (27.3%)	837 (18.6%)	12 (8.3%)
Secondary school, >2 yrs	254,367 (17.5%)	227,904 (17.6%)	25322 (16.1%)	1,105 (24.6%)	36 (25.0%)
University education	513,961 (35.4%)	460,247 (35.6%)	52458 (33.4%)	1,224 (27.2%)	32 (22.2%)
Unknown[a]	100,036 (6.9%)	90,691 (7.0%)	8738 (5.6%)	573 (12.8%)	34 (23.6)
**Income (Euro), mean, SD**	29,257±80,790	29,616±84,138	26,624±46,454	18,426±19,776	15,065±17,589
**Early retirement pension, N (%)**	60,378 (4.2%)	53,220 (4.1%)	6,778 (4.3%)	374 (8.3%)	6 (4.2%)
**Born in Sweden, N (%)**	1,193,374 (82.1%)	1,054,641 (81.6%)	134,663 (85.7%)	3,950 (87.9%)	120 (83.3%)
**Diagnoses, N (%)**					
Myocardial infarction	28,390 (2.0%)	24,747 (1.9%)	3,609 (2.3%)	32 (0.7%)	2 (1.4%)
Stroke	19,179 (1.3%)	16,408 (1.3%)	2,739 (1.7%)	30 (0.7%)	2 (1.4%)
Diabetes	112,797 (7.8%)	98,521 (7.6%)	14,001 (8.9%)	264 (5.9%)	11 (7.6%)
Hypertension	396,572 (27.3%)	343,103 (26.6%)	52,814 (33.6%)	635 (14.1%)	20 (13.9%)
Kidney failure	11,332 (0.8%)	9,700 (0.8%)	1,592 (1.0%)	38 (0.8%)	2 (1.4%)
COPD[#]	13,184 (0.9%)	11,064 (0.9%)	2,103 (1.3%)	16 (0.4%)	1 (0.7%)
Cancer	60,895 (4.2%)	51,803(4.0%)	8,992 (5.7%)	97 (2.2%)	3 (2.1%)

[a]Education and income was registered for individuals born earlier than October 2005. SD = standard deviation.

[#] Chronic obstructive pulmonary disease

© 2021 Nordström P, et al. *JAMA Internal Medicine.*

eTable 4. Risk of Covid-19 Infection in Families with Two-Four Members. Cox regression was used to calculate hazard ratios (HR) with the number of immune members in each family as exposure, and the outcome of Covid-19 infection in non-immune family members. Analyses were performed separately for families with 2-4 members, using families with no immunity as reference. Immunity was defined as single dose of vaccine, and individuals with two doses of vaccine or a previous infection were excluded.

Families with two relatives

Number of individuals with immunity in each family	Incident Covid-19 cases, (%)	HR	95% CI
None (N=1,069,442)	41,058 (3.8%)	1	(ref)
One (N=129,151)	2,464 (1.6%)	0.53	0.51-0.55

Families with three relatives

Number of individuals with immunity in each family	Incident Covid-19 cases, (%)	HR	95% CI
None (N=187,878)	26,034 (13.9%)	1	(ref)
One (N=23,794)	1,714 (7.2%)	0.51	0.48-0.53
Two (N=3,883)	181 (4.7%)	0.34	0.29-0.39

Families with four relatives

Number of individuals with immunity in each family	Incident Covid-19 cases, (%)	HR	95% CI
None (N=34,440)	14,949 (43.4%)	1	(ref)
One (N=4,203)	791 (18.8%)	0.37	0.35-0.40
Two (N=610)	59 (9.7%)	0.18	0.14-0.24
Three (N=144)	10 (6.9%)	0.12	0.07-0.23

© 2021 Nordström P, et al. *JAMA Internal Medicine*.
